# Supplementary material for: The Role of Adiponectin and Leptin in Fibro-Calcific Aortic Valve Disease: A Systematic Review and Meta-Analysis
Source: Biomedicines. 2024 Sep 2;12(9):1977. doi: 10.3390/biomedicines12091977 (PMC11428218; doi:10.3390/biomedicines12091977)
Supplement: Supplementary file 1 [file biomedicines-12-01977-s001.zip › biomedicines-3097108-supplementary.pdf]

SUPPLEMENTARY MATERIALS

The Role of Adiponectin and Leptin in Fibro-Calcific Aortic Valve Disease: A Systematic Review and Meta-Analysis

Veronika A. Myasoedova [1,*], Francesca Bertolini [1], Vincenza Valerio [1], Donato Moschetta [1], Ilaria Massaiu [1], Valentina Rusconi [1], Donato De Giorgi [1], Michele Ciccarelli [2], Valentina Parisi [3] and Paolo Poggio [1,4,*]

1 Centro Cardiologico Monzino IRCCS, 20138 Milan, Italy; francesca.bertolini@ccfm.it (F.B.); vincenza.valerio@ccfm.it (V.V.); donato.moschetta@ccfm.it (D.M.); ilaria.massaiu@ccfm.it (I.M.); valentina.rusconi@ccfm.it (V.R.); donato.degiorgi@ccfm.it (D.D.G.)
2 Department of Medicine, Surgery and Dentistry, University of Salerno, 84084 Fisciano, Italy; mciccarelli@unisa.it
3 Department of Translational Medical Sciences, Federico II University, 80138 Naples, Italy; valentina.parisi@unina.it
4 Department of Biomedical, Surgical and Dental Sciences, University of Milan, 20122 Milan, Italy
* Correspondence: veronika.myasoedova@ccfm.it (V.A.M.); paolo.poggio@ccfm.it (P.P.); Tel.: +39-02-5800-2748 (V.A.M.); +39-02-5800-2853 (P.P.)

SUPPLEMENTARY TABLES

Supplementary Table S1. Quality assessment of the included studies using the Newcastle–Ottawa scale.

Author, year	SELECTION				COMPARABILITY	OUTCOME			
	Representativeness of the Exposed Cohort	Selection of the Non-Exposed Cohort	Ascertainment of Exposure	Demonstration That Outcome of Interest Was Not Present at Start of Study		Assessment of Outcome	Enough Follow-Up Time for Outcomes to Occur	Adequacy of Follow-Up of Cohorts	Quality
Hansen 2023	**	*	*	-	*	*	-	*	7
Saracevic 2020	*	*	*	-	*	*	-	-	5
Lofti 2019	*	*	*	-	*	*	-	*	6
Liu 2019	**	**	*	-	*	*	-	*	8
Rosa 2017	*	*	*	-	*	*	-	*	6
Mizia-Stec 2017	*	*	*	-	*	*	-	*	6
Cabuk 2015	*	*	*	-	*	*	-	*	6
Cucuk Ipek 2013	*	*	*	-	*	*	-	*	6
Kolasa-Trela, 2011	*	**	*	-	*	*	-	*	7
Glader 2003	*	**	*	-	*	*	-	*	7

Supplementary Table S2. Characteristics of subjects included in the meta-analysis.

Author, year	Patient, n	CTRL, n	Age, years	Male sex, %	HT, %	DM, %	BMI, kg/m^2	CAD, %	TC, mg/dL	LDL-C, mg/dL	HDL-C, mg/dL	TG, mg/dL
**Hansen 2023**	309	650	62.6	51.1	84.9	-	26.4	59.5	242	-	-	--
**Saracevic 2020**	30	60	63.9	74.4	88.9	45.6	-	-	-	-	-	-
**Lofti 2019**	41	72	65.7	44.2	-	-	24.9	-	151	79.2	51	122
**Rosa 2017**	43	129	70.2	55.8	-	32.0	27.6	75.0	-	-	-	-
**Liu 2019**	200	197	66.6	60.2	70.8	31.2	-	78.3	151	87	43	141
**Mizia-Stec 2017**	65	24	68.5	41.1	78.7	26.7	29.2	42.7	187	113	46	150
**Cabuk 2015**	68	40	67.6	58.3	64.8	30.6	31.4	26.9	194.9	-	47	139
**Cucuk Ipek 2013**	58	24	70.3	52.4	63.4	19.5	27.1	42.7	190	117	43	128
**Kolasa-Trela 2011**	74	74	57.9	77.0	57.4	-	27.9	-	190	112	50	133
**Glader 2003**	101	101	71.3	59.4	-	-	25.3	-	-	-	-	-

CTRL: control; HT: hypertension; DM: diabetes mellitus; BMI: body mass index; CAD: coronary artery disease; TC: total cholesterol; LDL-C: low-density lipoprotein cholesterol; HDL-C: high-density lipoprotein cholesterol; TG: triglycerides.

SUPPLEMENTARY FIGURES

A

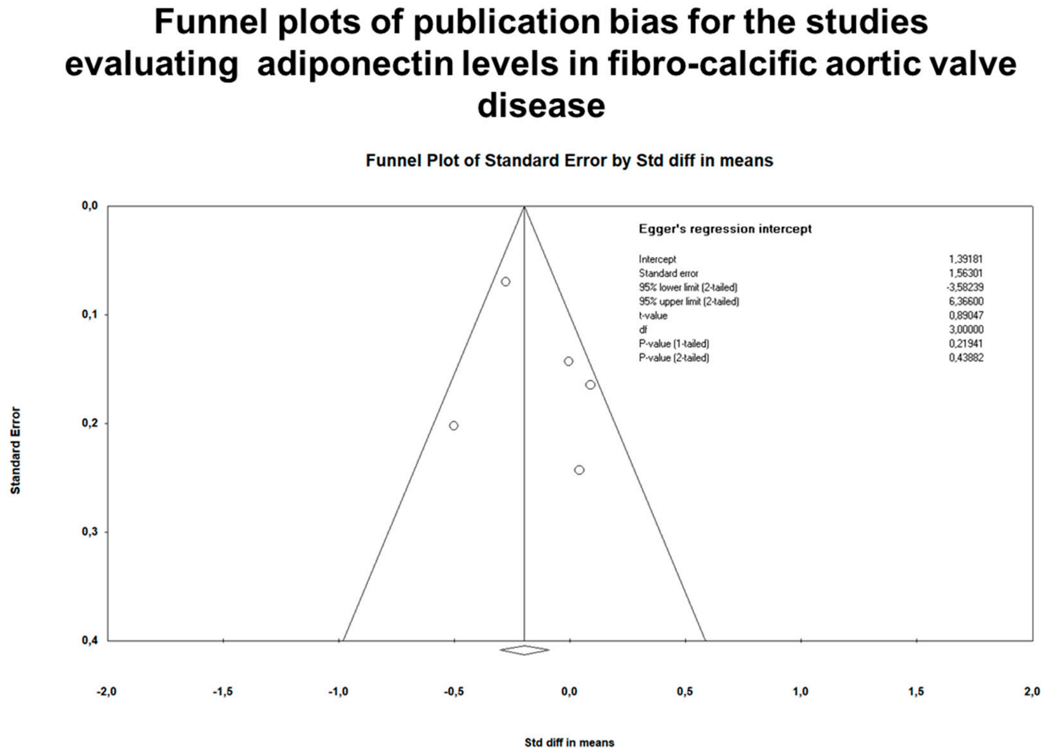

B

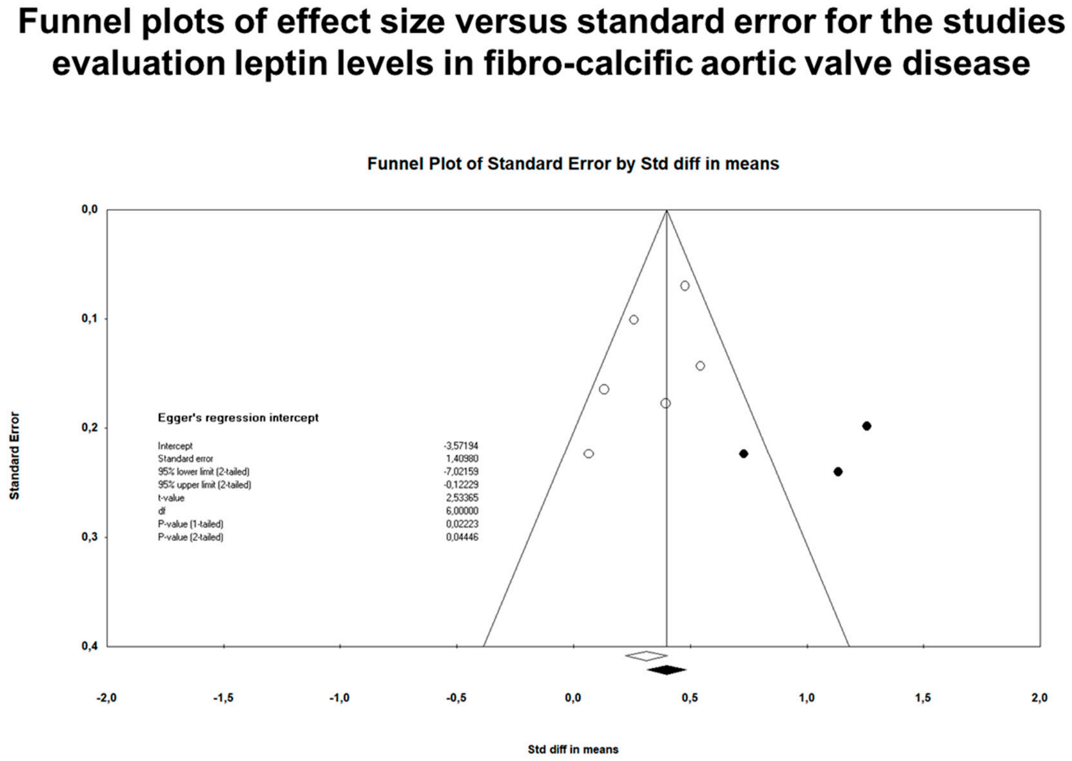

Supplementary Figure S1. Funnel plots of effect size vs. standard error for the included studies, evaluating the differences in adiponectin (**A**) and leptin (**B**) levels between patients with fibro-calcific aortic valve disease and controls. The dots represent single studies, while the diamonds are the overall standardized mean difference (Std diff in means). The white filling refers to real studies, while the black filling relates to Duval and Tweedie's trim-and-fill method.

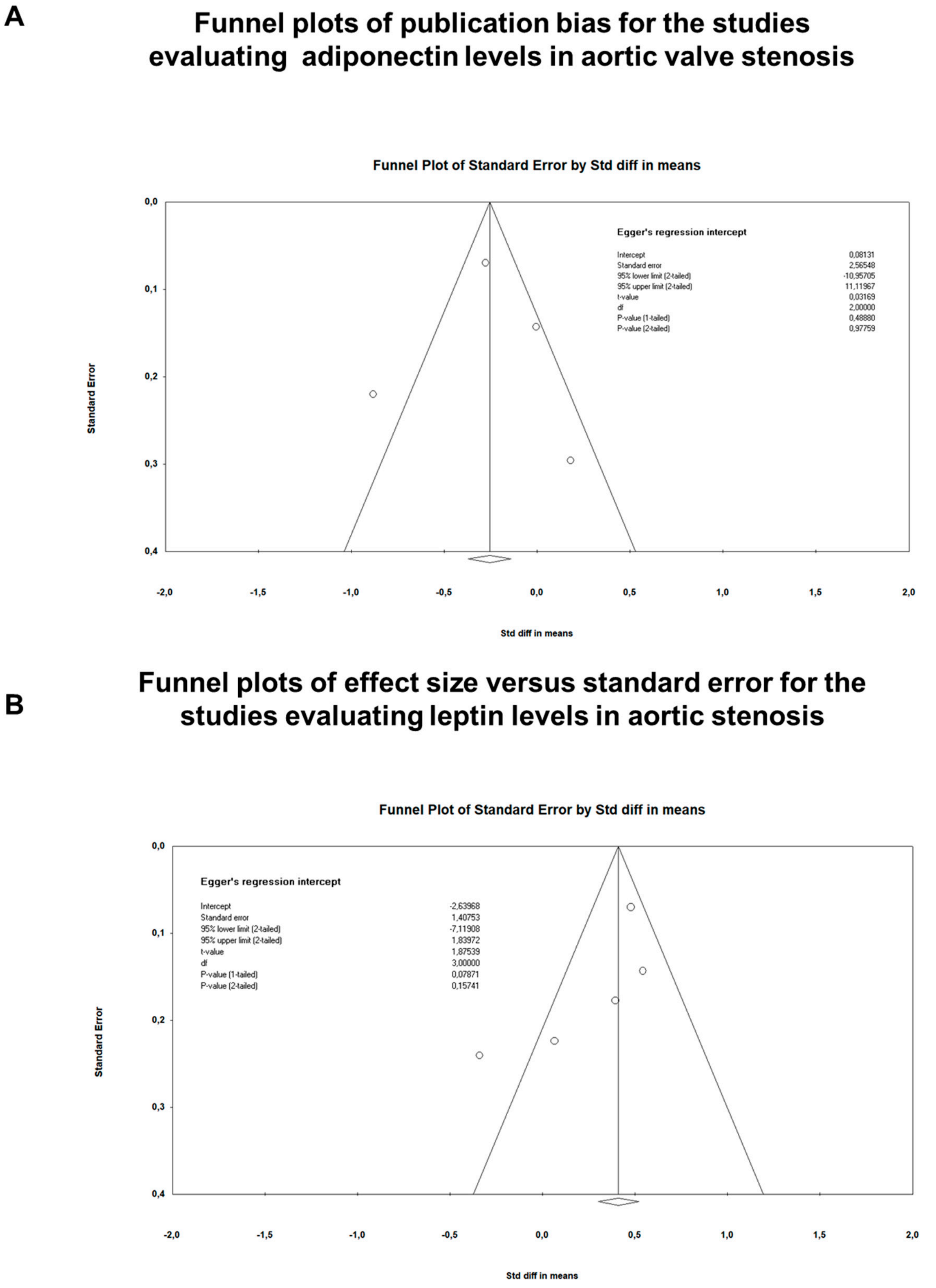

Supplementary Figure S2. Funnel plots of effect size vs. standard error for the included studies, evaluating the differences in adiponectin (**A**) and leptin (**B**) levels between patients with severe aortic stenosis and controls. The dots represent single studies, while the white diamonds are the overall standardized mean difference (Std diff in means).

A

Forest plots of leptin levels in men and women with fibro-calcific aortic valve disease

Study name	Sample size: Men	Sample size: Women	Statistics for each study: Std diff in means	Standard error	Variance	Lower limit	Upper limit	Z-Value	p-Value	Relative weight	Relative weight
Hansen 2023_Controls	334	314	-7,405	0,220	0,048	-7,837	-6,974	-33,628	0,000	20,01	
Hansen 2023_AS	155	153	-5,045	0,233	0,054	-5,502	-4,588	-21,649	0,000	20,00	
Glader 2003_Controls	60	41	-2,775	0,281	0,079	-3,327	-2,224	-9,863	0,000	19,94	
Glader 2003_AS	60	41	-1,383	0,225	0,051	-1,823	-0,942	-6,151	0,000	20,01	
Lofti 2019_All	68	45	-0,583	0,196	0,038	-0,967	-0,199	-2,975	0,003	20,04	
			-3,438	1,321	1,745	-6,027	-0,849	-2,603	0,009		

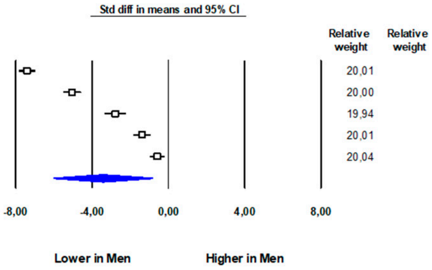

B

Funnel plots of effect size versus standard error for the studies evaluating leptin levels in men and women with fibro-calcific aortic valve disease

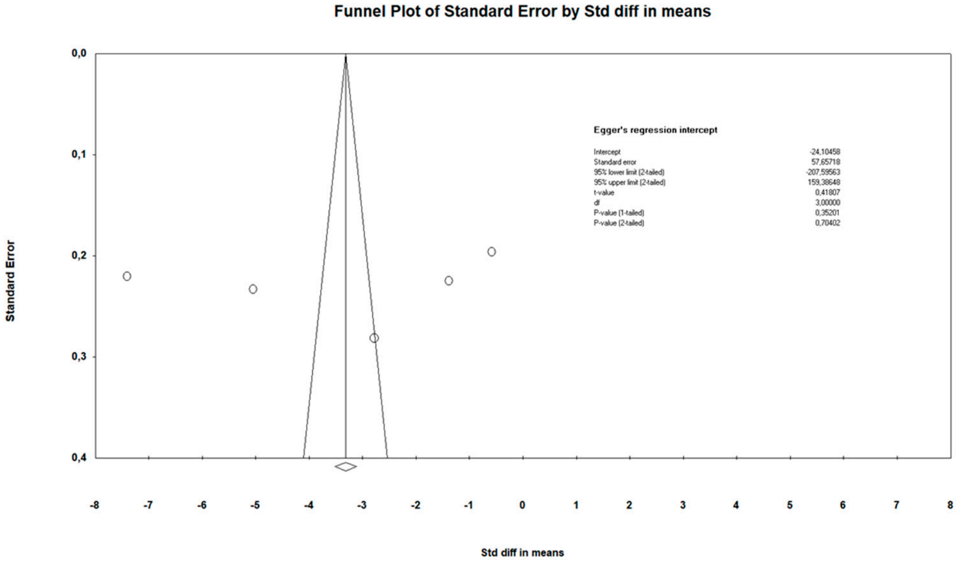

Supplementary Figure S3. Leptin levels in men and women with fibro-calcific aortic valve disease. (**A**) Forest plot of the leptin levels in men and women with fibro-calcific aortic valve disease presented as standardized differences in means (SMDs). The diamond represents the estimated overall effect, while the squares represent each study with 95% CI. (**B**) Funnel plot of effect size vs. standard error for the included studies, evaluating the differences in leptin levels between men and women with and without severe aortic stenosis. The dots represent single studies, while the diamond is the overall standardized mean difference (Std diff in means).
